# Effect of GnRH analogues for pituitary suppression on oocyte morphology in repeated ovarian stimulation cycles

**DOI:** 10.5935/1518-0557.20190050

**Published:** 2020

**Authors:** Bianca Ferrarini Zanetti, Daniela Paes de Almeida Ferreira Braga, Amanda Souza Setti, Assumpto Iaconelli Jr., Edson Borges Jr.

**Affiliations:** 1 Fertility Medical Group, São Paulo, SP - Brazil; 2 Instituto Sapientiae – Centro de Estudos e Pesquisa em Reprodução Humana Assistida, São Paulo, SP - Brazil

**Keywords:** pituitary suppression, GnRH, oocyte dysmorphisms, oocyte morphology, ICSI

## Abstract

**Objective:**

To compare the effect of pituitary suppression regimens on oocyte morphology in consecutive ICSI cycles of the same patients.

**Methods:**

Data was obtained from 200 matched consecutive intracytoplasmic sperm injection (ICSI) cycles performed in 100 couples undergoing the first cycle with the GnRH agonist and the following cycle with the GnRH antagonist regimen, from January 2010 to August 2016, in a private university-affiliated IVF centre. The effects of the pituitary suppression type on oocyte morphology were assessed by multivariate General Linear Models.

**Results:**

Mean interval between cycles was 185.32±192.85 days. Maternal age, body mass index, and total FSH dose administered were similar in both patients' cycles. Antagonist cycles presented lower incidence of dark cytoplasm (0.69±3.28% *vs.* 4.40±17.70%, *p*=0.047), Smooth endoplasmic reticulum (SER cluster (4.37±11.62% *vs.* 7.36±17.17%, *p*=0.046), and ZP defects (6.05±14.76% *vs.* 11.84±25.13%, *p*=0.049). Similar numbers of follicles retrieved oocytes, and mature oocytes were observed between the GnRH groups, as well as the fertilisation rate, number of obtained embryos, high-quality embryo rates, and the clinical outcomes.

**Conclusion:**

GnRH antagonist's inhibitory effect on the ovaries in consecutive ICSI cycles results in improved oocyte maturity and morphology, despite similar laboratory and clinical outcomes, compared to the GnRH agonist treatment.

## INTRODUCTION

Oocyte quality is an important factor that influences the developmental and implantation potential of derived embryos (Lazzaroni-Tealdi *et al*., 2015). In assisted reproduction, heterogeneity in oocyte quality is quite common. Differences between oocytes are enhanced by controlled ovarian stimulation (COS), which is unable to promote follicular recruitment and growth in a uniform fashion (Baerwald *et al*., 2012). Oocyte heterogeneity has implications for essential aspects of assisted reproduction, such as embryo selection for transfer, oocyte and embryo cryopreservation, cycle efficiency, and treatment safety. This justifies why, in preparation for intracytoplasmic sperm injection (ICSI), oocytes are observed to detect, if not characteristics positively associated with oocyte quality, at least dysmorphisms possibly incompatible with embryo viability (Dal Canto *et al*., 2017).

Typical evaluation of the oocyte quality is based on its nuclear maturation status and presence of specific extra and intracytoplasmic morphologic features (Rienzi *et al*., 2012). A high-quality metaphase II (MII) oocyte is defined as one with a clear moderately granular cytoplasm, a small perivitelline space, and a clear zona pellucida (Veeck, 1988). Its evaluation can be made under a microscope after oocytes have been denuded and the nuclear maturation stage has been determined.

Between 60-70% of the oocytes recovered after controlled ovarian stimulation (COS) exhibiting one morphological variation or more (de Cássia S Figueira *et al*., 2010), which may be explained by the fact that COS allows the maturation of oocytes, which under natural conditions would become atretic and regress (Jungheim *et al*., 2015). Morphological variations of oocytes may also result from intrinsic factors, such as age, or from extrinsic factors, such as stimulation protocols, culture conditions, and nutrition (Balaban & Urman, 2006).

Oocyte dysmorphisms are generally classified as being either intracytoplasmic, e.g., vacuoles, inclusions, smooth endoplasmic reticulum, central cytoplasmic granularity, and diffuse cytoplasmic granularity, or extracytoplasmic, e.g., abnormalities in zona pellucida, increased perivitelline space, first polar body fragmentation, and abnormal oocyte shape (Van Blerkom, 1990).

It has been hypothesised that some morphological variations may affect the developmental competence and implantation potential of the derived embryos (Balaban & Urman, 2006; Ebner *et al*., 2006; Balaban *et al*., 2008), but their effect has not yet been conclusively defined.

A meta-analysis of intracytoplasmic sperm injection (ICSI) cycles indicated that among eleven features evaluated, the presence of large polar body (PB), large perivitelline space (PVS), refractile bodies, and vacuoles negatively affected the oocytes fertilisation (Setti *et al*., 2011). Additionally, the importance of oocyte morphology evaluation came out by the recommendation to not inseminate oocytes with smooth endoplasmic reticulum clusters, due to its correlation with impaired blastocyst implantation (Setti *et al*., 2016), and poor neonatal outcomes (Harton *et al*., 2011).

Pituitary suppression, achieved by the administration of gonadotropin-releasing hormone (GnRH) agonists or antagonists, is an essential step of COS protocols to prevent premature luteinizing hormone (LH) surge and ovulation (Shrestha *et al*., 2015). In addition to the GnRH regulatory role on the hypothalamic-pituitary-gonadal axis, the GnRH receptor is also expressed in extrapituitary tissues such as follicular granulosa cells in the ovary, being involved in follicular growth, follicular steroidogenesis, and oocyte meiotic maturation (Takekida *et al*., 2003). Therefore, the use of GnRH analogues may impact not only hypothalamus-pituitary regulation but also oocyte development. The correlation between the types of pituitary suppression with oocyte morphology is still not elucidated.

Therefore the goal for the present study was to compare the effect of pituitary suppression regimens on oocyte morphology in consecutive ICSI cycles of the same patients.

## MATERIAL AND METHODS

### Experimental design, patients, and inclusion and exclusion criteria

This retrospective case-control within-subject study included data from 1,860 oocytes obtained from 200 matched ICSI cycles performed in 100 couples with tubal infertility from January 2010 to August 2016, in a private university-affiliated in vitro fertilisation (IVF) centre. The inclusion criteria were as follows: couples undergoing their first ICSI cycle with the GnRH agonist regimen and the following ICSI cycle with the GnRH antagonist regimen. Ovarian stimulation in both cycles had to be achieved by the administration of recombinant FSH. Only cycles with fresh embryo transfer at day five were included. The exclusion criteria were as follows: couples undergoing ICSI with vitrified/thawed or donated oocytes, surgical sperm retrieval, vitrified/thawed embryo transfer, or pre-implantation genetic diagnosis or screening.

The influence of the type of pituitary suppression (GnRH agonist or antagonist) on the incidence of oocyte morphological abnormalities (dysmorphisms) in each ICSI cycle was evaluated. The dysmorphisms were recorded as the incidence of (i) intracytoplasmic granulation clusters, (ii) SER clusters, (iii) vacuoles, (iv) dark cytoplasm, (v) perivitelline space (PVS) granules, (v) large PVS, (vi) zona pellucida (ZP) defects, and (ix) polar body (PB) fragmentation.

All patients signed a written informed consent form, and the study was approved by the Research Ethics Committee of the Faculdade de Medicina de Jundiaí (approval # 410/2012).

### Controlled ovarian stimulation

In the GnRH agonist regimen, pituitary suppression was achieved by the administration of leuprolide acetate (Lupron Kit; Abbott SA Societé Française des Laboratoires, Paris, France), initiated on the 22^nd^ day of the previous cycle. Ovarian stimulation was achieved with a daily dose of 225 IU recombinant FSH (rFSH, Gonal F; Serono, Geneva, Switzerland) for seven days. On day eight of ovarian stimulation, follicular development was monitored by transvaginal ultrasound, and the rFSH dose was reduced to 150 IU until the day of the ovulation trigger.

In the GnRH antagonist regimen, ovarian stimulation was achieved with a daily dose of 225 IU recombinant FSH (rFSH, Gonal F; Serono, Geneva, Switzerland) for seven days. On day eight of ovarian stimulation, follicular development was monitored by transvaginal ultrasound, and the GnRH antagonist was started (Cetrotide^®^; Merck KGaA, Darmstadt, Germany) in the presence of at least one follicle ≥14 mm. The rFSH dose was reduced to 150 IU until the day of the ovulation trigger.

The next steps are the same for the two regimens. When adequate follicular growth was confirmed by transvaginal ultrasound (≥3 follicles measuring ≥17 mm in diameter), recombinant hCG (r-hCG, Ovidrel^®^, Serono) was administered to trigger final follicular maturation. The oocytes were collected at 36±2 h through transvaginal ultrasound ovum pick-up.

### Oocyte preparation

Retrieved oocytes were maintained in culture media (Global for fertilisation, LifeGlobal, Guilford, USA) supplemented with 10% protein (LGPS, LifeGlobal Guilford, USA) and covered with paraffin oil (Paraffin oil P.G., LifeGlobal Guilford, USA) for 2-3 h before cumulus cell removal. The surrounding cumulus cells were removed after exposure to hyaluronidase (80 IU/ml, LifeGlobal Guilford, USA) followed by mechanical removal by a hand-drawn Pasteur pipette (Humagen Fertility Diagnostics, Charlottesville, USA). Oocyte morphology was assessed by highly trained embryologists, using an inverted Nikon Diaphot microscope with a Hoffmann modulation contrast system (Nikon Eclipse TE 300, Tokyo, Japan) under 400× magnification, just before sperm injection (5 h after retrieval). Mature oocytes were used for the ICSI.

### Intracytoplasmic sperm injection

Sperm selection was analysed at 400× magnification using an inverted Nikon Eclipse TE 300 microscope (Nikon, Tokyo, Japan). The injection was performed in a micro-injection dish prepared with 4 µl droplets of buffered medium (Global w/HEPES, LifeGlobal, Guilford, USA) and covered with paraffin oil on a heated stage at 37.0ºC±0.5ºC on an inverted microscope. Fertilisation was confirmed by the presence of two pronuclei and the extrusion of the second polar body approximately 16 h after ICSI.

### Embryo quality and embryo transfer

Embryos were morphologically evaluated on days two, three, and five of development using Nikon Eclipse TE 300 microscope (Nikon, Tokyo, Japan) under 400× magnification.

High-quality cleavage-stage embryos were defined as those with all of the following characteristics: 3-5 cells on day 2 or 8-10 cells on day 3, <15% fragmentation, symmetric blastomeres, and absence of multinucleation. Embryos lacking any of these characteristics were considered to be of low quality. The blastocysts rate was defined as the number of embryos that reached the blastocyst stage at day five by the number of 2PN embryos.

Embryos were placed in a 50 µL drop of culture medium (Global, LifeGlobal, Guilford, USA) supplemented with 10% protein supplement and covered with paraffin oil in a humidified atmosphere under 7.5% CO_2_ at 37ºC for five days. Embryo transfer was performed on the fifth day of development using a soft catheter with transabdominal ultrasound guidance. One to three embryos were transferred per patient, depending on embryo quality and maternal age.

### Clinical follow-up

A pregnancy test was performed 10 to 12 days after embryo transfer. All women with a positive test received a transvaginal ultrasound scan after two weeks. Clinical pregnancy was diagnosed when the foetal heartbeat was detected. Implantation rates were calculated per patient. Pregnancy rates were calculated per embryo transfer. A miscarriage was defined as a pregnancy loss before 20 weeks.

### Data analysis and statistics

The sample size calculation using G*Power 3.1.7 (Franz Faul, Universität Kiel, Germany) suggested that 84 couples would be enough to demonstrate a 20% effect with an 80% power and 5% significance level in multivariate general linear models, considering the incidence of oocytes with abnormal morphology as the primary endpoint.

The oocyte dysmorphisms were recorded as the percentage of oocytes affected per cycle. The effects of the type of pituitary suppression (GnRH agonist *vs.* antagonist) on oocyte dysmorphisms, response to COS, and laboratory and clinical outcomes were assessed by Multivariate General Linear Models. The statistical analyses were performed using SPSS Statistics 21 (IBM, New York, New York, USA). Data are expressed as the mean±standard deviation for continuous variables, while percentages are used for categorical variables.

## RESULTS

The mean interval between cycles was 185.32±192.85 days (range 24-954 days). In the first cycle, under the GnRH agonist regimen, 977 oocytes were retrieved, and in the subsequent cycle, with the GnRH antagonist, 883 oocytes were obtained.

Antagonist cycles presented lower incidence of dark cytoplasm (0.69±3.28% *vs.* 4.40±17.70%, *p*=0.047), SER cluster (4.37±11.62% *vs.* 7.36±17.17%, *p*=0.046), and ZP defects (6.05±14.76% *vs.* 11.84±25.13%, *p*=0.049) (Table 1).

**Table 1:** Descriptive analysis of patients’ characteristics, COS outcomes, and oocytes dysmorphisms, for the first ICSI cycle with the GnRH agonist regimen, and the following ICSI cycle with the GnRH antagonist regime

	GnRH Agonist	GnRH Antagonist	*P*
Maternal age (years)	35.59±3.39	35.97±3.54	0.444
Maternal BMI (kg/m^2^)	23.27±3.51	23.15±3.19	0.813
Total FSH dose (IU)	2532.94±90.76	2484.37±90.79	0.706
***COS outcomes***			
Oestradiol peak (pg/ml)	2232.41±2345.78	1731.75±1524.74	0.128
Follicles (n)	14.52±10.53	13.89±10.60	0.485
Oocytes (n)	9.87±7.04	8.83±6.19	0.453
Oocyte Yield (%)	69.70	66.01	0.713
Metaphase II oocyte (n)	7.17±5.53	6.77±4.82	0.647
Mataphase I oocyte (n)	1.39±1.77	1.03±1.65	0.105
Prophase I oocyte (n)	1.30±1.87	1.03±1.78	0.757
Mature oocytes rate (%)	73.82	79.3	0.342
Oocyte dysmorphisms*			
Granulation cluster (%)	4.04±14.52	5.61±16.91	0.483
Dark cytoplasm (%)	4.40±17.70	0.69±3.28	0.047
sERC (%)	7.36±17.17	4.37±11.62	0.046
Vacuoles (%)	5.80±15.76	6.50±15.00	0.746
Perivitelline granules (%)	45.36±32.31	51.24±34.32	0.213
Large PVS (%)	24.08±29.21	24.51±29.24	0.917
ZP dysmorphism (%)	11.84±25.13	6.05±14.76	0.049
PB fragmentation (%)	33.10±26.30	32.30±27.75	0.677

* Controlled for the presence of other oocyte dysmorphisms.

Note: COS—controlled ovarian stimulation; BMI—body mass index; FSH—follicle-stimulating hormone; IU—international unit; sERC—smooth endoplasmic reticulum cluster; PVS—perivitelline space; ZP—zona pellucida; PB—polar body.

There were no differences in maternal age and BMI, total FSH dose administered, and estradiol level between cycles. Similar numbers of follicles, retrieved oocytes, and mature oocytes were observed between the GnRH groups (Table 1).

The fertilisation rate, number of obtained embryos, high-quality embryo rates, and clinical outcomes were similar between the GnRH groups (Table 2).

**Table 2:** Descriptive analysis of laboratory and clinical outcomes for the first ICSI cycle with the GnRH agonist regimen and the following ICSI cycle with the GnRH antagonist regime

	GnRH Agonist	GnRH Antagonist	*p*
***Laboratory outcomes***			
Fertilization rate (%)	80.31	79.72	0.262
Embryos obtained (n)	5.78±0.24	6.05±0.25	0.432
Embryos transferred (n)	2.19±0.15	1.93±0.14	0.219
High-quality embryo at day two (%)	22.13±2.31	27.84±2.31	0.081
High-quality embryo at day three (%)	40.27±2.99	45.29±2.94	0.229
Blastocyst development (%)	42.21±4.55	47.81±4.81	0.433
***Clinical outcome***			
Endometrial thickness	11.39±2.31	11.01±1.84	0.114
Implantation rate	18.21±37.93	25.89±44.48	0.121
Cycle’s cancelation rate	9.00	16.00	0.189
Pregnancy rate	32.07	31.53	0.542
Miscarriage rate	9.52	5.00	0.519

## DISCUSSION

As a fundamental step in COS, GnRH analogues are used for suppression of endogenous pituitary gonadotropin release, prevention of premature ovulation, and recovery of a larger number of oocytes, leading to increased chance of pregnancy. Our data suggest an increased oocyte maturation rate and a better oocyte morphology when the pituitary blockage is performed by GnRH antagonists compared to GnRH agonists in consecutive ICSI cycles.

While the GnRH agonists induce an initial secretion of FSH and LH, followed by potent down-regulation and inhibition of the pituitary-gonadal axis, the GnRH antagonists act promptly, reversibly suppressing the pituitary, thereby avoiding FSH and LH release (Kumar & Sharma, 2014). Whether oocyte development is affected by the pituitary suppression regimes is still not elucidated.

Although it was suggested that oocyte dysmorphisms repeatedly occur in cycles from the same patients (Meriano *et al*., 2001), in our study the frequency of some dysmorphisms changed substantially between cycles, suggesting that COS protocols may affect the quality of retrieved oocytes.

A higher proportion of mature oocytes was obtained in the GnRH antagonist cycles after the first COS with GnRH agonists, in agreement with a previous report (Cavagna *et al*., 2011). Although the proportions of prevalent dysmorphisms, such as PVS granules and PB fragmentation, were similar between cycles, decreases of 85% in the incidence of dark cytoplasm, 62% in ZP defects, and 53% in SER clusters were noted in antagonist cycles. In agreement with this, Otsuki *et al*. (2004) found that SER aggregations were more common in short GnRH agonist protocols as compared with antagonist protocols.

Conversely, Murber *et al*. (2009) found a significantly higher incidence of cytoplasmic changes in the GnRH antagonist regime when compared with the agonist. Moreover, Cota *et al*. (2012) did not find any difference in terms of oocyte morphology when the two protocols were compared. Differences in the study design, in the populations, and the analysis methods may explain the controversial results.

The presence of dark cytoplasm has been related to increased risk of obtaining poor quality embryos in cycles with donor oocytes (Ten *et al*., 2007). Nevertheless, a prospective study reported similar fertilisation and embryo quality rates in GnRH agonist cycles with or without dark cytoplasm (Esfandiari *et al*., 2006). The substantial alteration in the incidence of this dysmorphism suggests that pituitary suppression with GnRH agonists may be involved with its emergence; however, the impact of dark cytoplasm on subsequent clinical outcomes has not been described.

Defects of the ZP, including its composition, colour, shape, and thickness, have been extensively associated with ultrastructural alterations in oocytes, lower fertilisation, implantation, and clinical pregnancy rates (Shi *et al*., 2016). To our knowledge, to date, only one study investigated the correlation between GnRH analogues and ZP quality, in which antagonist protocols resulted in oocytes with lower birefringence of the inner zona layer, which is a negative predictor for blastocyst formation (Ebner *et al*., 2010). The correlation between GnRH analogues and the incidence of ZP defects is still inconsistent, but the present study showed that antagonist cycles presented a lower incidence of this dysmorphism.

The presence of SER clusters has been correlated with impaired blastocyst formation and implantation, higher biochemical pregnancy rate, lower clinical pregnancy rate, and poor neonatal outcomes (Setti *et al*., 2016). These poor outcomes were observed even though transferred embryos were derived from SER cluster-negative oocytes of the same cohort, which highlights the importance of this dysmorphism. High oestradiol levels have been attributed as a possible cause of SER cluster formation (Otsuki *et al.,* 2004). In fact, we observed a trend towards a higher oestradiol level on the hCG day in the GnRH agonist cycles, which could be associated with the higher SER cluster incidence in these cycles. The reduction of oestradiol level after the GnRH antagonist regimen may, in this case, result in improved oocyte quality (Kol, 2000).

No associations between the type of pituitary suppression and fertilisation or subsequent embryo development were observed in this study, however, nearly a 30% increase in high-quality embryo rates at day two and more than a 10% increase in the high-quality embryo rates at day three were noticed in GnRH antagonist cycles, despite the lack of statistical significance. Other studies comparing agonist and antagonist protocols also described that there was no difference in fertilisation and early embryo development, although a slightly higher embryo quality in the antagonist group was observed (Lai *et al*., 2013).

It has been previously demonstrated that GnRH antagonists, used for pituitary suppression, directly influence the development of extrapituitary tissue, including the endometrium (Hernandez, 2000), therefore, it could be argued that the role of the GnRH antagonists on embryo implantation involves besides embryo development, the endometrial receptivity. Nevertheless, in the present study, endometrial thickness and clinical outcomes were similar between both pituitary suppression regimens, similar to what has been corroborated by others (Xavier *et al*., 2005; Detti *et al*., 2008).

The reason why the same clinical results were noted when pituitary blockage was performed either by the GnRH agonist or antagonist, even when an increased maturation and better morphology was observed in oocytes retrieved from the GnRH agonist cycles, is not completely clear. The role of the oocyte in embryo development is well documented, while the contribution of the sperm cell to the embryo has not been fully appreciated. However recent reports have highlighted the importance not only of the male gamete (Dahm, 2005; Machtinger *et al*., 2016) but also of the different male epigenetic marks, such as DNA methylation (Siklenka *et al*., 2015), and post-translational modification (PTMs) of histones and protamines (Castillo *et al*., 2014; Siklenka *et al*., 2015) to the zygote.

Moreover, it is well known that implantation depends not only on proper embryo development, but also on the acquisition of a receptive endometrium, and an adequate dialogue between the two (Dominguez *et al*., 2003). In fact, in the present study, no significant differences in endometrial thickness could be noted when the pituitary blockage protocols were compared.

Although no significant differences could be noted, clinical results, especially the pregnancy rate, should be analysed with caution, since the study included repeated cycles in which positive or negative outcomes were achieved in the first cycle. Positive results in a first attempt would bias the results of the second attempt.

A few limitations in this study should be noted: (i) despite that oocyte morphology had been assessed by highly trained embryologists, inter-observer variation cannot be ruled out; (ii) none of the cycles had the cohort of oocytes 100% affected by any of the analysed dysmorphisms, and (iii) the GnRH antagonist protocol has been shown to improve pregnancy outcome in patients with a history of previous treatment failures (Lai *et al*., 2013).

## CONCLUSION

Oocyte dysmorphisms may be influenced by the type of GnRH administered in stimulated cycles, and the GnRH antagonist regimen is associated with higher rates of mature oocyte and lesser incidence of oocyte dysmorphisms, despite similar laboratory and clinical outcomes, compared to the GnRH agonist treatment.
